# Desmoplastic Infantile Ganglioglioma: *cytologic findings and differential diagnosis on aspiration material*

**DOI:** 10.1186/1742-6413-2-1

**Published:** 2005-01-11

**Authors:** Oluwole Fadare, M Rajan Mariappan, Denise Hileeto, Arthur W Zieske, Jung H Kim, Idris Tolgay Ocal

**Affiliations:** 1 Department of Pathology, Yale University School of Medicine, New Haven, CT, USA

## Abstract

**Background:**

Desmoplastic infantile ganglioglioma (DIG) is a rare WHO Grade I tumor of infancy that is characterized by large volume, superficial location, invariable supratentoriality, fronto-parietal lobe predilection and morphologically, by an admixture of astroglial and neuroepithelial elements in a desmoplastic milieu. With over 50 cases described, the histologic and radiographic spectrum of DIG has been well-characterized. The superficial location of DIGs may render them greatly amenable to preoperative assessment utilizing aspiration cytology; however, the cytologic features of this rare tumor have only been reported once previously.

**Case Presentation:**

We present herein cytomorphologic findings from the intraoperative aspiration of a typical case of DIG diagnosed in a 1-year-old male. As evaluated on a single liquid-based preparation, the specimen showed low cellularity and was comprised predominantly of a population of dispersed (occasionally clustered) large neuronal cells with eccentrically located hyperchromatic nuclei (which were occasionally binucleated) and abundant unipolar cytoplasm. Rare smaller astroglial cells were intermixed. Despite the tumor's characteristic desmoplastic histologic appearance, no stromal fragments were identified on the aspiration material.

**Conclusions:**

A differential diagnosis is presented and analyzed in detail and it is concluded that when these large neuronal cells are encountered in an aspirate of a brain mass in a child, a combination of clinical, radiologic and immunohistochemical parameters can eliminate most of the differential possibilities.

## Background

The clinicopathologic features of 11 examples of a distinctive pediatric tumor designated *desmoplastic supratentorial neuroepithelial tumors of infancy *(also known as desmoplastic infantile ganglioglioma, [DIG]) were originally described by Vandenberg et al in 1987 [1]. Since that seminal report, at least 40 additional cases have been described, such that the clinical, radiologic and histopathologic features of this tumor are now well-defined. An uncommon tumor that constituted less than 0.04% of all central nervous system (CNS) tumors in one series [2], DIG is classified as a Grade 1 tumor in the World Health Organization (WHO) classification of CNS tumors [3]. They most commonly occur in children less than 18 months of age [1] who typically present with symptoms related to an intracranial mass effect [3]. DIGs are generally of large size, are solid to cystic, show a predilection for the frontal and parietal cerebral lobes, and are typically superficially located with at least focal attachment to the overlying dura [1-3]. The superficial location of DIGs may render them greatly amenable to preoperative assessment utilizing aspiration cytology. However, there is a dearth of information on the cytomorphologic features of these tumors [4]. To contribute information of possible utility in their pre-operative or intra-operative assessment, we report herein cytomorphologic features associated with a typical case of DIG.

### Case Presentation

**Figure 1:**
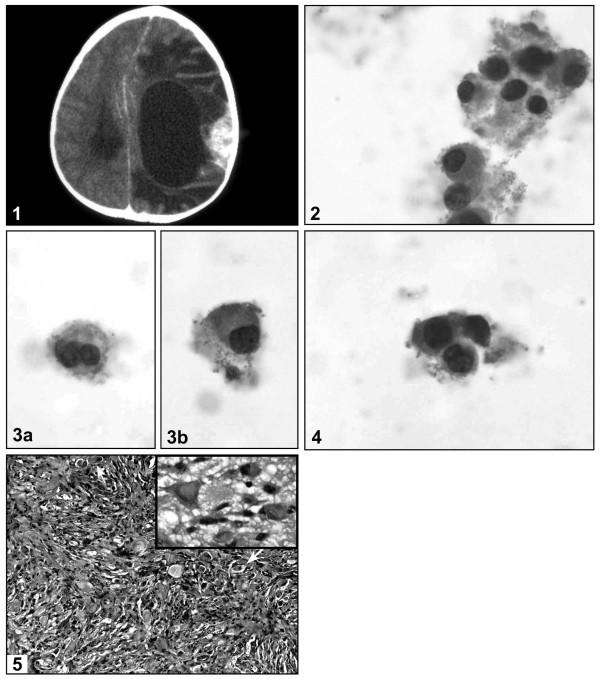
Radiologic, cytologic and morphologic appearance of the tumor. **1**: This computed-tomographic scan of the patient's cerebral mass shows a large cystic mass with peripheral enhancement at the solid portion which attached to the overlying dura; **2**: In addition to scattered individual cells, variably sized clusters of neuronal cells were identified, all composed of cells with eccentrically located, occasionally binucleated hyperchromatic nuclei and abundant unipolar cytoplasm [original magnifications ×400]; **3**: Occasional neuronal cells were binucleated (**3a**) while others showed bland nuclear features (**3b**) [original magnifications ×400]; **4**: Scattered astroglial cells with more convoluted nuclear contours and less cytoplasm were also present. [original magnifications ×400]; **5**: Typical histologic appearance of desmoplastic infantile ganglioglioma, showing scattered ganglion cells in a desmoplastic and fibroblastic, vaguely storiform background (original magnification ×200, inset ×400)

A one-year-old boy was noted to have a striking increase in head circumference as compared to a previous measurement. Neurological examination and developmental status were normal at that point. Within 2 weeks, the patient deteriorated rapidly, with poor mobilization, feeding and verbalization. He was brought to the emergency room where an emergent computed tomographic scan showed a large left hemispheric cerebral mass (Figure 1-1) with an underlying cystic component and a more superficial area of bright enhancement; the rest of the brain showed massive edema. He was emergently admitted and within 24 hours, a gross resection of the tumor was carried out. At surgery, following a parietal craniotomy, 50–60 cc of straw colored fluid was aspirated from the cystic component through a taut dura. After the excision of the dura, the bright area of enhancement previously noted was an area of tumor attachment to the dura in the parietal region. Otherwise, the tumor showed a well-demarcated interface with the subjacent normal brain parenchyma and a complete gross resection was achieved. A follow-up magnetic resonance image at 12 months post-surgery showed no evidence of tumor recurrence. Functionally, the patient was felt to have a mild right hemiparesis and some probable language delay, but otherwise showed no neurological deficits.

### Materials and Methods

For cytology, a slide was prepared from 50–60 cc of straw-colored fluid utilizing the ThinPrep^® ^2000 Automated Slide Processor (Cytyc, Boxborough, MA) according to the manufacturer's instructions. For the tumor specimen, approximately 10 × 6.5 cm of fragmented gray and white cerebral tissue was received and entirely processed routinely: tissue sections were fixed in 10% neutral buffered formalin, processed, embedded in paraffin, sectioned to 4 μ-thick sections and stained with hematoxylin and eosin, Nissl stain and reticulin. The immunohistochemical profile of the tumor was evaluated on 4 μ thick, formalin-fixed, deparaffinized sections using a DAKO Autostainer (Carpinteria, CA, USA) based on the avidin-biotin-peroxidase complex with antibodies ki-67 (dilution 1:320, DakoCytomation Corp, Carpinteria, CA), synaptophysin (dilution 1:600, DakoCytomation) and glial fibrillary acid protein [GFAP] (dilution 1:10, DakoCytomation).

### Pathologic findings

As evaluated on a single liquid-based preparation, the specimen showed low cellularity and was comprised predominantly of a population of dispersed (occasionally clustered) large neuronal cells (~70 μm diameter each) with round eccentrically placed uniform hyperchromatic nuclei, undulating and slightly convoluted nuclear membranes, and abundant unipolar granular cytoplasm (ganglion cells) (figures 1-2 and 1-3). Occasional cells were binucleated (figure 1-3a). A spectrum in the degree of nuclear membrane irregularities was noted, with most cells displaying irregular features as described above, while other cell showed bland nuclear features (figure 1-3b). However, all displayed nuclear polarity to the cytoplasm. Rare smaller cells interpreted as astroglial cells were interspersed between the larger cells. The latter cells showed nuclear hyperchromasia, more prominent irregularities in their nuclear membranes and a smaller cytoplasmic rim. (figure 1-4). Overall, significant proportions of both cellular populations showed varying degrees of degenerative changes manifested as lack of clear delineation of nuclear and cytoplasmic borders and a loss of nuclear detail. Several clusters were composed of large cells, and in these clusters, constituent large cells showed less cytoplasm but retained a unipolarity in relation to the nuclei and their nuclear features were identical to those of the more predominant population of large cells. Additionally, scattered foamy histiocytes were present. A finely granular background material consistent with necrosis was present, but there was no distinct neurofibrillary material.

Vascular structures or stroma were not present. Histologically, the tumor was partially attached to the dura and was present in the subarachnoid space. The bulk of the specimen was a variably cellular desmoplastic component whose predominant constituent cells were elongated spindle cells arranged in a reticulin-rich, storiform pattern (figure 1-5). At higher magnification, ganglion-type cells (Nissl stain positive) with 1–4 round nuclei, prominent nucleoli, and abundant unipolar cytoplasm were present (Figure 1-5, inset). Immature or abortive ganglion cells with enlarged single nuclei and markedly irregular nuclear membranes were rare but identifiable morphologically. Less "differentiated" aggregates of cells with hyperchromatic nuclei and minimal cytoplasm, as has been well-described in DIGs [1-3], were present. Mitotic figures were rare and small foci of necrosis were limited to the less differentiated component. Immunohistochemical stains for synaptophysin was positive in the ganglion cells only. GFAP was positive in astroglial elements within the desmoplastic regions; the latter was negative for synaptophysin. The ki-67 labelling index was 11.3% (evaluated in the area of greatest density of positive staining cells).

## Discussion

The clinical presentation, radiographic appearance and histopathologic features of this case are entirely consistent with those described for desmoplastic infantile ganglioglioma [1-3]. There has been a significant evolution in the understanding of this rare tumor since its original description in 1987 [1]. DIGs are typically large supratentorial tumors that, at least as observed radiographically in one patient, are initially solid then become cystic [5]. Although this tumor is considered a grade 1 tumor based on the histopathologic features of cases described prior to the publication of the WHO monograph in 2000, at least one report has since documented anaplastic features in a case of DIG, which was ultimately fatal [6]. However, follow-up has generally been favorable following complete resection in the reported cases of DIG, with a median post-surgical interval of 8.7 years without metastases or recurrence in one series of 14 patients [2]. Additionally, in some cases, spontaneous regression of tumor following subtotal tumor resection has been documented [7,8].

The distinctive clinical features of DIG, being a typically superficially located tumor occurring in young children (with potentially unclosed fontanelles), may render them particularly amenable to pre-operative assessment using aspiration cytology. In addition, familiarization of practitioners with the cytopathologic features of DIG may be useful because 1) With the aforementioned cases of DIG regressing after subtotal resection [7,8], it might be unnecessary to aggressively resect these tumors to negative margins, and a preoperative aspiration diagnosis of DIG will be helpful in the neurosurgical planning and 2) In their intra-operative assessment, imprint cytopathology may potentially be more diagnostic than histopathology. However, to our knowledge, the cytologic features of DIG have been documented only once previously [4]. In that report, Hasegawa et al [4] reported aspiration and imprint cytology findings in two cases of DIG. Two distinct cellular populations were identified, a predominant population of small to intermediate sized astroglial cells and "a few" large cells with round nuclei, prominent nucleoli and profuse cytoplasm that was unipolar to the nuclei in all their illustrations. In the current case, the reverse was found, with the predominant cells being an identical population of large cells with round nuclei, prominent nucleoli and abundant unipolar cytoplasm, and only rare unequivocal astroglial cells. Most of the analysis of the aforementioned report [4] was on the imprint smears, and although a mixture of small and large cells were also identified on the aspiration smear, there was no stated assessment or low-power illustration of the relative ratio of small to large cells on the latter. In the current case, a cell-block for immunohistochemical confirmation of the nature of two-cell population was unavailable; however, the larger cells were positive for neurofilament (confirming their neuronal nature) while the smaller cells were positive for GFAP (confirming their astroglial nature) in the report of Hasegawa et al [4].

The finding of large cells with eccentrically located nuclei and abundant unipolar cytoplasm in an aspiration specimen of a cerebral mass occurring in a young person should generate a differential diagnosis that includes DIG, atypical teratoid/rhabdoid tumor (AT/RT), dysembroplastic neuroepithelial tumor (DNT), ganglioglioma, supratentorial primitive neuroectodermal tumour (PNET) with ganglionic differentiation (ganglioneuroblastoma), anaplastic large cell lymphoma and pleomorphic xanthoastrocytoma. In our opinion, clinical features as well as immunohistochemical analysis can significantly help reduce the likelihood for most of the aforementioned entities. The distinction of DIG from AT/RT is probably of the greatest prognostic significance, since in contrast to DIG, AT/RT is a highly malignant tumor that is almost uniformly fatal [9]. Although AT/RT occurs in infants or young children, most cases occur in the posterior fossa, in contrast to DIGs, which are invariably supratentorial [1-3]. Radiographically, AT/RT are typically not distinctly cystic, although necrosis may impart an irregularly cystic appearance.

Immunohistochemically, the rhabdoid cells of AT/RT co-express vimentin and epithelial membrane antigen [10], in contrast to the ganglion cells of DIG. However, rhabdoid cells may rarely express neurofilament, an immunophenotypic overlap with DIG.

Morphologically, the distinct cytoplasmic borders, "inclusion-like" cytoplasmic globule and overall dense eosinophilia of the cytoplasm of rhabdoid cells, in conjunction with the aforementioned clinicopathologic parameters, may help in their distinction from ganglion cells of DIG [9,10]. The separation of DIG from other tumors containing true ganglion cells based on cytomorphology alone would probably pose the greatest difficulty. These tumors include DNT, ganglioglioma, and supratentorial PNET with ganglionic differentiation; all have a predilection for, or at least may potentially occur in children. In addition to ganglion cells, cytomorphologic features of DNT include oligodendroglial-like cells arranged in lobules and neurons in abundant extracellular mucin or neurofibrillary material [11,12]; these findings were neither identified in the 2 cases of DIG reported by Hasegawa et al [4], nor the current case. The distinction of gangliogliomas form DIG based on cytomorphology alone may be impossible even in the presence of a significant stromal component on the aspirate. Gangliogliomas, like DIG may be solid to cystic and show desmoplasia, although in contrast to DIG, they have a predilection for the temporal lobe and most commonly occur in an age group slightly older than is typical for DIG [13]. However, it should be noted that conventional gangliogliomas and DIG may exist on a morphologic spectrum, and a case with morphologic features of both entities has been described [14]. Supratentorial PNET with ganglionic differentiation (ganglioneuroblastomas) also show significant clinicopathologic overlap with DIG, as they are supratentorial, occur in young children, may be cystic and may show desmoplasia [15]. Cerebral ganglioneuroblastomas are extremely rare, and in the absence of treatment-related cytodifferentiation, will show a significant neuronal component of smaller cells. However, the cytomorphologic features of pure ganglioneuroblastoma have not been well-characterized.

Other less likely differential considerations include pleomorphic xanthoastrocytoma and anaplastic large cell lymphoma (ALCL), both of which may contain large cells with eccentrically located nuclei and abundant cytoplasm. The temporal lobe predilection, lack of neuronal differentiation, presence of xanthomatous cells, smaller tumor size and older age of patients with pleomorphic xanthoastrocytoma should permit an easy distinction of the large cells in this tumor from those of DIG. Primary brain ALCL is exceedingly rare and generally occurs in older individuals, with a mean age of 29 years in one series [16]. Immunoreactivity for CD30, ALK and CD45 in ALCL and absence of similar immunoreactivity in DIG should facilitate a distinction in rare cases that occur in very young children.

When the cytomorphologic findings of DIG are described in more cases, it is likely that the cytologic spectrum will mirror the histologic heterogeneity of this tumor. For example, astroglial cells predominated in the two cases of Hasegawa et al [4] while the ganglion cells predominated in ours. In addition, it is conceivable that an aspirate would only capture the immature neuroepithelial cells which frequently characterizes DIG. Nonetheless, it is concluded that when the large neuronal cells are encountered in an aspirate of a brain mass in a child, a combination of clinical, radiologic and immunohistochemical parameters can eliminate most of the differential possibilities.

## Competing interests

The author(s) declare that they have no competing interests.

## Authors' contributions

All authors made substantial contributions to the intellectual content and/or presentation of the manuscript. ITO (cytologist), diagnosed the cytopathological aspects of the case and co-supervised the entire project. JHK (neuropathologist), diagnosed the histological aspects of the case and co-supervised the project. OF wrote the initial version of the manuscript. MRM, DH and AWZ collected pathological, clinical and/or photographical information and revised the manuscript.
